# Moving to malaria elimination in Cape Verde

**DOI:** 10.1186/1475-2875-11-S1-O9

**Published:** 2012-10-15

**Authors:** Julio Monteiro Rodriguez, Jean Olivier Guintran, Carolina Gomes, Socé Fall, Aafje Rietveld, Richard Cibulskis, Robert D Newman, Rossitza Kurdova-Mintcheva

**Affiliations:** 1 Ministry of Health, Praia, Cape Verde; 2 Global Malaria Programme, World Health Organization, Geneva, Switzerland; 3 WHO country Office, Praia, Cape Verde; 4 WHO Regional Office for Africa, Brazzaville, Congo

## Background

This case study examines the history of malaria in Cape Verde and presents details of the interventions implemented over the last 60 years with the aim of disease elimination. Trends in the malaria situation and programme policies and operations over time were evaluated and lessons captured to assist other countries in making well-informed decisions regarding malaria elimination. The case study is a part of a joint series of malaria elimination case studies conducted by the WHO Global Malaria Programme and the University of California, San Francisco, Global Health Group.

## Materials and methods

A review of published literature in English, French and Portuguese, of relevant documents and guidelines of the Ministry of Health, documents from WHO archives, and archives from the “Instituto de Medicina Tropical” (IMT) in Lisbon was conducted. Epidemiological, programmatic, demographic, social, and other relevant data was extracted, analysed and evaluated.

## Results

Before the 1950s, Cape Verde was meso-endemic (*Plasmodium falciparum*, *P.vivax* and *P.malaraie* were detected) with annual incidence rates around 100 per 1000 population. The first malaria elimination campaign was initiated in 1953. Using IRS with DDT as a main strategy, and applying additionally antilarval activities and active case finding, malaria transmission was interrupted in 1967; and in 1969 control interventions were stopped. The unique vector *Anopheles arabiensis* had been eradicated from all islands but Santiago.

In 1973, transmission reappeared in Santiago, culminating in a major epidemic with 844 cases and 13 deaths in 1978. Widespread IRS operations were resumed for 5 years and transmission was again interrupted (1983-1986). A new epidemic occurred in 1987-88 in Santiago Island and localised IRS operations were reactivated for 2 years; the country has not been able to invest sufficient resources to operate a new elimination plan.

Low level malaria transmission continued on Santiago and, in 2003, reappeared on the island of Boa Vista. The annual parasite incidence for the whole archipelago has remained below 0.3 per 1000 population, exceeding 0.5 per 1000 population on Santiago Island only once, in 2000. Over the last 20 years, activities have been restricted to passive case finding and investigation with case-based surveillance on Santiago, and to early detection of imported cases elsewhere. Unfortunately, in 2006, an unexpected 8 malaria deaths occurred.

Formulated in 2007, the National Health Policy sets out the strategy for durably eliminate malaria by 2020. The Government developed a National Strategic Plan 2009-2013 with an integrated approach and the following main strategies: drug policy change to artemisinin-based combination therapy (ACT) for *P. falciparum*; case detection among all febrile patients with positive travel history; full reporting of microscopically confirmed cases; case and foci investigation; vector control – including focal larval control where appropriate, and IRS. National funding for malaria has been increased; in 2011, a 5-year Global Fund grant was secured to support the programme transition to elimination.

## Conclusions

In Cape Verde, malaria transmission has already been interrupted twice within the last 50 years, confirming that elimination is technically feasible; the challenge is now sustainability. The importance of preventing malaria reintroduction should be fully taken into account if elimination is to be achieved.

